# Combined Effect of an Active AgIon^®^ Absorbent Pad and a Chitosan Coating on the Preservation of Fresh Beef

**DOI:** 10.3390/foods13091387

**Published:** 2024-04-30

**Authors:** Dimitrios Komodromos, Daniel Sergelidis, Ioannis Amvrosiadis, Michael G. Kontominas

**Affiliations:** 1 Department of Chemistry, University of Ioannina, GR-45110 Ioannina, Greece; dkomodro@vet.auth.gr; 2 Department of Veterinary Medicine, Aristotle University of Thessaloniki, GR-54124 Thessaloniki, Greece; dsergkel@vet.auth.gr (D.S.); ambros@vet.auth.gr (I.A.)

**Keywords:** active packaging, chitosan, microbiological/chemical/sensory analysis, beef shelf life

## Abstract

In the present study, the combined effect of an AgIon^®^ antimicrobial absorbent (Ζ) pad and a chitosan coating (C) on the preservation of fresh beef stored aerobically at 5 °C was investigated. Microbiological, physicochemical, and sensory attributes were monitored for up to 10 days of storage. The microbiological data indicated that the C and chitosan coating plus absorbent pad (CZ) treatments were the most efficient in reducing total viable counts (TVC) by 4.09 and 3.53 log cfu/g compared to the control W and Z treatments on day 4 of storage (*p* < 0.05). An analogous reduction in the counts of the other microbial groups monitored was recorded. pH values were ca. 5.7 for treatments W and Z and 5.45 for treatments C and CZ on day 4 of storage (*p* < 0.05). The total volatile basic nitrogen (TVB-N) values remained <20 mg/100 g for all treatments on day 4 and for treatments C and CZ on day 10 of storage. The total color difference values decreased (*p* < 0.05) during storage for treatments W and Z, but remained constant for treatments C and CZ. Based on sensory, microbiological and physico-chemical data, beef shelf life was ca ^# + 3 days for samples W and Z and at least 10 + 3 days for samples C and CZ. Between the two antimicrobial treatments, chitosan was considerably more effective than the AgIon^®^ antimicrobial absorbent pad, which showed practically no antimicrobial activity in direct contact with beef meat.

## 1. Introduction

Fresh meat is highly susceptible to spoilage due to microbial growth and chemical and biochemical activity due to its specific composition and pH, resulting in the deterioration of its sensory properties and thus its rejection by consumers [1]. The main bacteria associated with refrigerated beef spoilage are *Pseudomonas* spp., *Brochothrix thermosphacta*, *Shewanella putrefaciens*, *Carnobacterium* spp., Enterobacteriaceae, *Lactobacillus* spp. and *Leuconostoc* spp. [1,2]. Furthermore, beef provides a favorable environment for the growth of pathogens including *Staphylococcus aureus,* Clostridia, and *Aeromonas* spp., etc., which are responsible for food safety problems [2]. Το ensure the high quality and adequate safety of meat, packaging in the form of modified atmosphere packaging (MAP), vacuum packaging (VP), active and intelligent packaging, etc., or in combination with natural preservatives has been successfully used to substantially extend product shelf life [3,4].

Among contemporary packaging technologies, active packaging (AP) is defined by the European Commission Regulation (EC) No 450/2009 [5] as that which provides functions beyond the inert barrier and traditional protection of the contained product from the external environment. AP technology is based either on scavenging or emitting systems. Scavenging systems are added inside the package to remove substances (e.g., oxygen, carbon dioxide, moisture, odors, ethylene gas), while emitting systems are used to generate substances (i.e., carbon dioxide, ethanol, flavors, antimicrobials, antioxidants, etc.) during product storage and distribution [4]. Antimicrobial packaging technologies that have been used include the use of ethanol, carbon dioxide, silver ions, essential oils, etc. [6].

Commercial silver particle-based antimicrobial trays, films, and coated paper including Agion^®^ (AgIon technologies, Wakefield, MA, USA), Food-touch^®^ (MicrobeGuard Corp., Elk Grove Village, IL, USA), etc., have been used as food-contacting layers of laminated polymeric materials during the transport and storage of meat [7]. Such silver-based packaging materials have been provisionally approved as food-contacting materials in the EU and the USA, provided that silver migration into the foodstuff does not exceed the 0.001 mg/kg limit set by the EU [5].

Alternatively, antimicrobial films can be prepared using natural polymers exhibiting inherent antimicrobial properties. An example of these is chitosan.

Chitosan [b-(1,4)-2-amino-2-deoxy-D-glucopyranose] is an amino-poly-saccharide produced from chitin isolated from crustacean shells through deacetylation. Chitosan possesses remarkable functional properties, such as its biodegradability, film-forming capacity, non-toxic nature, and biocompatibility, as well as its antimicrobial and antioxidant activity [8]. It has been documented to inhibit the growth of Gram-positive and Gram-negative bacteria and fungi [9]. Potential applications of chitosan as a food biopreservative have been reported for various meat products, functioning against both food spoilage and pathogenic microorganisms [10,11,12]. Chitosan was classified as GRAS by the USFDA as early as 2001 [13].

There are several studies published in the literature on the preservation of fresh beef through coating with chitosan [2,14,15,16,17,18,19]. However, to the best of our knowledge, the use of a Food-touch^®^ silver-based antimicrobial absorbent pad in combination with chitosan has not been investigated in fresh beef meat preservation; this comprises the novelty of the present study. The investigation of the above combination was decided in order to show the comparative effect of each of the two antimicrobial treatments on the shelf-life extension of fresh beef. Thus, the aim of this study was to determine the effect of the Food-touch^®^ absorbent pad and a chitosan coating, applied individually or in combination, on the microbiological, chemical, and sensory properties of fresh beef during refrigerated storage.

## 2. Materials and Methods

### 2.1. Absorbent Pads

Food-touch^®^ multilayer liners consisting of an absorbent texturized paper containing a Ag+-substituted zeolite laminated onto a low-density polyethylene (LDPE) film were provided by MICROBEGUARD Co. (Elk Grove Village, IL, USA). The above material is based on AgIon^®^ antimicrobial technology approved for food contact use in the EU by EFSA [20].

### 2.2. Beef Samples

Fresh beef (*Longissimus dorsi*) was provided by a local butcher shop 48 h post slaughter. During the 48 h storage period, the meat was held at 1 °C. The meat was then transported to the laboratory in a foam polystyrene box in ice within 1 h. Subsequently, the meat muscle was cut into parallelepiped chunks of 150 ± 10 g each using a sterilized knife and placed on the food-contacting side of the absorbent pad. An identical Food-touch^®^ absorbent pad was used to cover the meat samples (two-side contact). Experimentation was commenced the following day upon the arrival of beef samples (72 h post slaughter).

### 2.3. Preparation of Chitosan Solution

Food-grade chitosan powder originating from shrimp shells of high molecular weight (>800 kDa, degree of deacetylation: 75%) was purchased from Sigma-Aldrich Chemie GmbH, Taufkirchen, Germany). A 1.0% (*w*/*v*) chitosan solution was prepared in 1% (*v*/*v*) acetic acid. In order to fully dissolve chitosan, the chitosan/acetic acid suspension was stirred on a magnetic plate with the aid of a magnetic stirring bar for 5 h at room temperature. Glycerol (0.75 mL/g of chitosan) was added as a plasticizer. Based on the volume change of the chitosan solution before and after the dipping of the meat, a concentration of 0.1% (*w*/*w*) chitosan was established. Before the dipping process, a pre-sterilized stainless-steel hook was attached to each chunk of meat to ensure the complete coating of the meat. After dipping, the samples were hung in a laminar flow, where they remained for 1 h at room temperature in order to completely dry the coating.

### 2.4. Sample Treatments

Four groups of samples were prepared: the first group comprised the control samples (without chitosan dip and Food-touch^®^ absorbent pad) (W); the second group comprised samples wrapped in the Food-touch^®^ absorbent pad (Z); the third group comprised samples dipped in a 1.0% (*w*/*v*) chitosan solution (C); and the fourth group comprised samples dipped in a 1.0% chitosan and subsequently wrapped in the Food-touch^®^ absorbent pad (CZ). After treatment, all samples were placed in polystyrene foam trays and covered with PVC cling film (Tiktas Ltd., Thessaloniki, Greece) with an oxygen permeability of 560 cm^3^ O_2_/m^2^.day.atm, maintaining an aerobic environment throughout storage. Samples were then stored in an LBI-150M refrigerator (Daihan Labtech Co., Namyangju, Republic of Korea) operating at 5 ± 0.5 °C. Sampling was carried out on days 0, 2, 4, 6, 8, and 10 of storage.

### 2.5. Microbiological Analysis

Microbial counts were determined as follows: 25 g meat samples were transferred aseptically into individual stomacher bags (Seward and Co., Easting Close Worthing West Sussex, UK) containing 225 mL of buffered peptone water solution (BPW, 0.1%). Samples were homogenized for 2 min by using a Lab Blender 400, Stomacher (Seward and Co., Ltd., Worthing, UK) at room temperature. Further serial decimal dilutions were prepared in BPW solution (0.1%) for each sample. Then, 0.1 mL of these serial dilutions of meat homogenates was spread on the surface of agar plates. Microbiological analyses included the total viable count (TVC) determined using plate count agar (PCA, LAB M, Lancashire, UK) after incubation at 37 °C for 2 days. Pseudomonads were determined on pseudomonas agar base (LAB M, Lancashire, UK) supplemented with the selective supplement CFC (cephalothin-fucidin-cetrimide, LAB M, Lancashire, UK) after incubation at 25 °C for 48 h. *Brochothrix thermosphacta* was determined on STAA agar base enriched with the selective supplement STAA (streptomycin sulphate-thallous acetate-actidione) (Oxoid, Basingstoke, UK) after incubation at 25 °C for 2 days and the subsequent checking of colonies with the oxidase test. Enterobacteriaceae were determined on violet red bile glucose agar (VRBGA, LAB M, Lancashire, UK) after incubation at 30 °C for 2 days. Finally, lactic acid bacteria (LAB) were determined on de Man–Rogosa–Sharpe agar (MRS, LAB M, Lancashire, UK) after incubation at 30 °C for 2 days. The enumeration of colonies was carried out according to APHA [21].

### 2.6. Physico-Chemical Analysis

The pH was recorded using a model HANNA pH model 211, pH-meter (HANNA Instruments, Smithfield, RI, USA) at ambient temperature. A total of 10 g of beef muscle was homogenized thoroughly with 90 mL of distilled water and the homogenate was used for pH determination. The determination of the color parameters L* (lightness), a* (redness-green color) and b* (yellow-blue color) was carried out using a model CR-410 colorimeter (Konica-Minolta Co., Tokyo, Japan) using a 1 cm diameter aperture, a C illumination source and a 0° viewing angle [22]. The colorimeter was previously calibrated using a white standard surface (L* = 97.06, a* = −0.14, b* = 1.93). For measurement purposes, the colorimeter head was in direct contact with the meat surface. In addition to the parameters L*, a* and b*, the total color difference (ΔE) indicating the change in sample color compared to the initial sample color was calculated. The determination of TVB-N was carried out according to method 981.10 of the AOAC for meat and meat products [23]. In brief, 10 g of meat was homogenized with 100 mL of distilled water in a beaker for 10 min followed by centrifugation for 10 min at 3000 rpm. The resulting solution was filtered and 5 mL of the filtrate along with 5 mL of 5% MgO, 10 mL of 10% (*w*/*v*) H_3_BO_3_, and a few boiling stones were transferred to a distillation flask. Finally, 5 mL of the resulting distillate was titrated within 5 min with a solution of 0.01 M HCl using an equilibrium mixture of 0.1 g of methyl-red and 0.05 g of methyl-blue in 100 mL of ethanol as an indicator. A blank sample consisting of distilled water and the titration indices was also titrated. The results were expressed in mg N_2_/100 g of the sample.

### 2.7. Sensory Evaluation

Sensory evaluation was carried out on both the raw and cooked meat samples. The sampling of raw meat took place at a frequency of every other day, while after sampling on the 4th and 10th day, samples were also subjected to sous-vide cooking (vacuum-packed in a Stomacher bag and immersed in hot water (T = 80 °C) for 20 min). The sensory panel consisted of 20 semi-trained judges in the 22–65 age group who consume meat on a regular basis. Evaluation was carried out in individual booths under controlled conditions of light and temperature. Panelists were asked to evaluate the appearance and odor of the raw meat and the color, odor, taste, and overall acceptability of cooked meat. For comparison purposes, panelists were served a control reference sample (stored at −20 °C and subsequently thawed) along with the test samples. Furthermore, the panelists were instructed to cleanse their palate by consuming a cracker and sipping water between evaluations of cooked samples. The hedonic scoring scale was 1–10, where 10 corresponded to the most liked sample and 1 corresponded to the least liked sample. A score of 5 was the lower acceptability limit.

### 2.8. Statistical Analysis

Experiments were replicated twice on different occasions with different meat samples originating from animals of the same age, breed and feeding protocol. Analyses were run in triplicate for each replicate (*n* = 2 × 3 = 6). Data were subjected to a two-way analysis of variance (ANOVA) using the STATGRAPHICS Centurion Statistical package (STATGRAPHICS Centurion XVI, version 16.1.11 for 32-bit Windows) (StatPoint Technologies Inc. Warrenton, Washington, DC, USA). Means and standard errors were calculated. Significance was defined at *p* = 0.05, and when F-values were significant at the *p* < 0.05 level, mean differences were separated by means of the least significant difference (LSD) procedure. Microbiological data were transformed into logarithms of the number of colony forming units (cfu/g).

## 3. Results and Discussion

### 3.1. Microbiological Analysis

**Figure 1:**
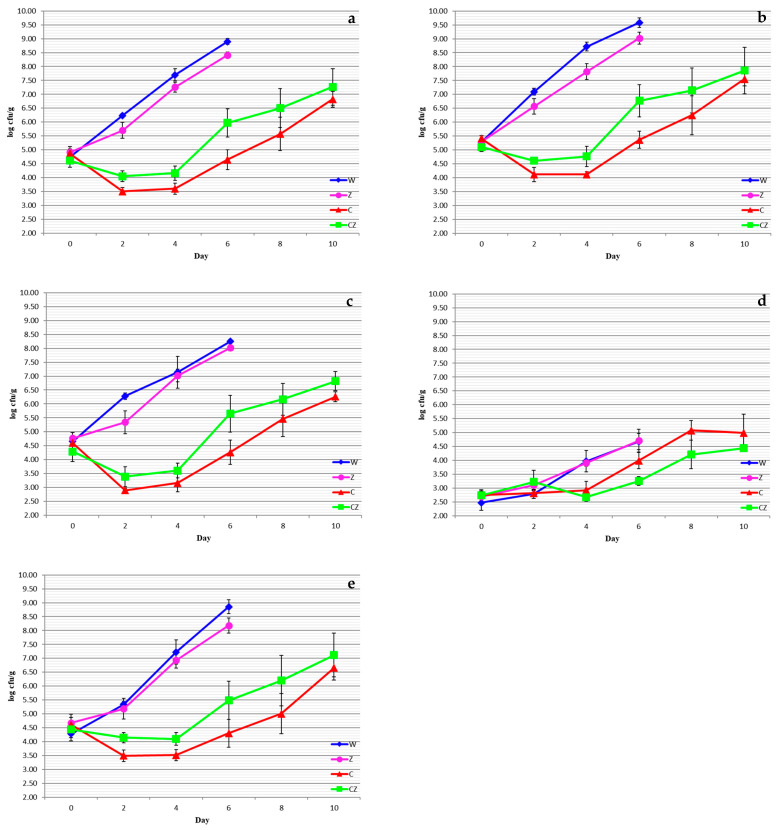
Microbial group counts of beef treatments as a function of storage time: (**a**) TVC, (**b**) pseudomonads, (**c**) *Brochothrix thermosphacta*, (**d**) lactic acid bacteria, (**e**) Enterobacteriaceae. W—control; Z—AgIon^®^ pad; C—chitosan; CZ—chitosan + AgIon^®^ pad.

TVC gives a quantitative estimate of the population of all microorganisms in a food sample capable of forming visible colonies. The majority of microorganisms present in fresh beef flesh, either as part of its natural microflora or as the result of cross contamination from other sources, are mostly aerobic microorganisms, and their population is an indicator of product microbiological quality. Figure 1a shows the changes in TVC of beef meat as a function of treatment and storage time.

As shown in Figure 1a, the initial value of TVC was 4.74 log cfu/g, indicative of good-microbiological-quality beef meat. TVC reached 7 log cfu/g, the upper microbiological limit for acceptable quality meat [24], on ca. day 3 (i.e., day 3 + 3 after slaughter) for the control samples (W), and day 3–4 (day 6–7 after slaughter) for the Ag^+^-substituted zeolite pad (Z), with no significant differences (*p* > 0.05) between the two treatments. Treatments C and CZ reached 7 log cfu/g on day 10 + 3 of storage, without significant differences (*p* > 0.05) between the two treatments. Significant differences (*p* < 0.05) were only noted between treatments W and Z and treatments C and CZ throughout storage. A first observation to be made is that the Ag^+^-substituted zeolite pad had a negligible effect in reducing TVC compared to the chitosan dip. Ιt has been suggested by Fernandez et al. [25] that the presence of natural chelating agents in food matrices reduces the antimicrobial power of silver and limits its applicability in food preservation technology. On the other hand, the antimicrobial activity of chitosan has been well documented owing to its positively charged macromolecules which interact with the negatively charged cell membrane of microorganisms altering cell permeability and blocking the transcription of RNA from DNA [26].

With regard to antimicrobial absorbent pads, similar results were reported by Castrica et al. [6], who studied the preservation of beef slices in one-side contact with an active pad soaked in an additive mixture comprising 30 to 50% b.w. of a polymeric cationic agent. It should be noted that the pad used in the above study did not contain Ag ions. Likewise, Fernandez et al. [25] reported that cellulose–nano-silver hybrid materials used in contact with beef eye of round cuts did not affect the populations of the major microbial groups (TVC, *Pseudomonas* spp. and lactic acid bacteria) determined in beef during the entire 11-day storage period.

With regard to the antimicrobial effect of chitosan, Chounou et al. [14] investigated the shelf-life extension of fresh ground meat stored at 4 °C using either chitosan (1% *w*/*w*) or an oxygen absorber or the combination of the two. Their results showed that microbial populations were reduced by 0.4–2.0 log cfu/g for a given sampling day, with a more pronounced effect being achieved using the combination of chitosan and the oxygen absorber. In another study, Hoa et al. [19] reported a 3.5 and 4 log cfu/g reduction in TVC in beef steaks dipped in a 2% chitosan solution and a 2% chitosan and 0.3% gallic acid solution, respectively, after 21 days of refrigerated storage. In a similar study by the same authors [15], beef steaks were dipped in a solution containing either 2% chitosan + 1 mM lauric acid or 2% chitosan + 3 mM lauric acid. Their results showed that with an initial TVC of 1.85 log cfu/g, TVC values reached 6.72, 4.42, 3.74, and 3.48 log cfu/g for the control sample, the sample coated only with chitosan, the sample coated with chitosan + 1 mM lauric acid and the sample coated with chitosan + 3 mM lauric acid, respectively, on day 21 of storage. Finally, Cheng et al. [16] coated beef slices with a chitosan-containing solution (0.5% chitosan + 0.2% ε-polylysine + 0.1% glutathione). Samples were stored for up to 21 days under refrigeration. Their results showed that the control sample reached 7 log cfu/g after 10 days of storage, while the TVC of treated steaks was ca. 3.7 log cfu/g on the same day. The individual effect of chitosan was not determined in the above study.

The pseudomonads are strictly aerobic bacteria comprising the major specific spoilage microorganisms of meat, chicken, and seafood [1]. Figure 1b shows the changes in the pseudomonads as a function of treatment and storage time. The pseudomonads followed a similar increasing trend to TVC, reaching 7 log cfu/g on day 2 of storage (i.e., day 5 after slaughter) for the control sample W, on day 3 for the zeolite-treated sample Z, and on day 10 for the chitosan-coated C and CZ samples. According to the ICMFS [24], a *Pseudomomas* spp. count of 7 log cfu/g is considered the upper acceptable limit for meat. As with TVC, there were no significant differences (*p* > 0.05) between treatments W and Z on day 2 and between treatments C and CZ on day 9 to 10 of storage. Significant differences (*p* < 0.05) were noted between treatments W and Z and treatments C and CZ throughout storage. With regard to antimicrobial absorbent pads, the results of the present study are in agreement with those of Castrica et al. [6] and Fernandez et al. [25], who reported no statistically significant effect of the antimicrobial pad on the *Pseudomonas* spp. count of fresh beef samples. With regard to the antimicrobial effect of chitosan beef meat, the present results are in general agreement with those of Chounou et al. [14], Hoa et al. [19], and Hoa et al. [15], who reported a substantial reduction in the *Pseudomonas* spp. count of fresh beef during storage.

*Brochothrix thermosphacta* is a facultative anaerobe and is considered, along with the pseudomonads, a spoilage specific microorganism for meats packaged aerobically or under modified atmosphere [27]. Figure 1c shows the changes in *Brochothrix thermosphacta* count in fresh beef during storage. The *B. thermosphacta* count increased sharply from an initial population of ca. 4.5 log cfu/g to one exceeding 7.0 log cfu/g on day 4 (i.e., day 7 after slaughter) for both the W and Z treatments, with no significant differences (*p* > 0.05) between the two treatments. The respective counts of *B. thermosphacta* were 6.3 and 6.8 log cfu/g for the C and CZ treatments on day 10 of storage, without significant differences (*p* > 0.05) between the two treatments. Thus, the increasing trend for *B. thermosphacta* was similar to that for TVC and the pseudomonads. With regard to the effect of the antimicrobial pad, the present results are in agreement with those of Castrica et al. [6], who reported a negligible effect of antimicrobial absorbent pads on *B. thermosphacta* counts. With regard to the effect of chitosan, the present results are in agreement with those of Chounou et al. [14] for fresh beef meat and Karakosta et al. [8] for fresh water buffalo meat.

LAB are fermentative facultative anaerobes which can be categorized as homo- or hetero-fermenters depending on the nature of the fermentation products [28]. They comprise part of meat’s natural spoilage microflora, mainly producing sour flavors and butter odors due to the production of lactic acid [29]. Figure 1d shows the changes in LAB as a function of treatment and storage time. The initial LAB count was ca. 2.6 log cfu/g, reaching 4.7 log cfu/g on day 6 of storage (i.e., day 9 after slaughter) for the W and Z treatments, while no significant differences (*p* > 0.05) were recorded between the two treatments. The LAB counts for the C and CZ treatments reached 5.0 and 4.5 log cfu/g, respectively, on day 10 of storage, with no significant differences (*p* > 0.05) between the two. The populations of LAB were relatively low as their growth was restricted by the strictly aerobic pseudomonads which dominated the meat microflora. Regarding the non-significant effect of the antimicrobial pad on the LAB populations, the present results are in agreement with those of Castrica et al. [6] and Fernandez et al. [25] for fresh beef cuts. Regarding the effect of the chitosan treatment, the results of the present study are in agreement with those of Chounou et al. [14], Duran and Kahve, [2], Hoa et al. [19], and Hoa et al. [15] for fresh beef cuts and those of Karakosta et al. [8] for water buffalo meat.

Finally, Enterobacteriaceae are facultative anaerobes which, along with TVC, total coliforms and *E. coli*, are considered as hygiene indicators in the food industry. They grow well both under aerobic and anaerobic conditions. The growth pattern of the Enterobacteriaceae was very similar to that of TVC (Figure 1e). The initial population of Enterobacteriaceae was ca. 4.5 log cfu/g and exceeded 7.0 log cfu/g on day 3–4 (i.e., day 6–7 after slaughter) for both the W and Z treatments, with no significant differences (*p* > 0.05) between the two treatments. The respective counts of the Enterobacteriaceae were 6.7 and 7.1 log cfu/g for the C and CZ treatments on day 10 of storage, without significant differences (*p* > 0.05) between the two treatments. With regard to the effect of the antimicrobial pad, the results of the present study are in agreement with those of Castrica et al. [6] for fresh beef cuts. Regarding the effect of the chitosan treatment, the results of the present study are in agreement with those of Chounou et al. [14] for fresh beef cuts and those of Karakosta et al. [8] for water buffalo meat.

The comparison of the differences in microbiological group populations determined between the control and the Ag^+^-substituted zeolite pad (in most cases less or equal to 0.5 log cfu/g), corresponding to a microbiological shelf-life extension of roughly one day, raises concerns regarding the actual antimicrobial activity of such commercial products. All three studies, including that of Fernandez et al. [25], the present one and that of Castrica et al. [6] working with beef meat, provide this same conclusion. Yet, Fernandez et al. [25] reported significant differences in microbial counts between the controls and the meat fluids absorbed in the antimicrobial pads used. This implies that there should be some kind of interaction between the pad and the foodstuff matrix that inhibits the antimicrobial activity of the absorbent pad. According to Matsamura et al. [30], the antimicrobial activity of the Ag^+^-substituted zeolite may be inhibited either by the high protein or high salt content of the food substrate. It is thus postulated that the direct contact between the pad containing the Ag^+^-substituted zeolite and the foodstuff (i.e., meat) does not allow for the necessary ‘moisture bridge’ required for the liberation of Ag^+^ from the zeolite matrix for the expression of the antimicrobial activity. This postulation is supported by the results of both Fernandez et al. [25], working with meat fluids absorbed by pads exhibiting a clear antimicrobial activity, and Lee et al. [31], working with meat broth.

### 3.2. Physico-Chemical Analysis

**Figure 2:**
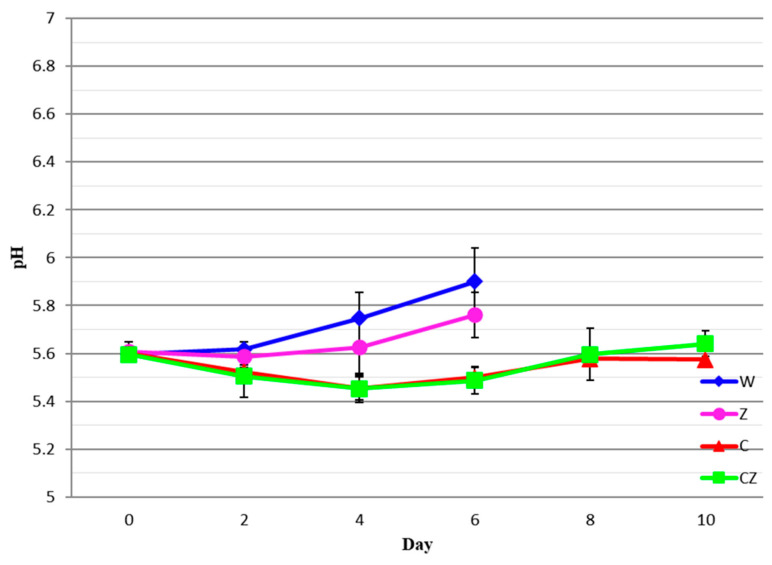
pH values of beef treatments as a function of storage time. W—control; Z—AgIon^®^ pad; C—chitosan; CZ—chitosan + AgIon^®^ pad.

The pH values for all meat treatments as a function of storage time are shown in Figure 2.

The initial pH for all treatments was ca. 5.60. On day 4 of storage (i.e., day 7 after slaughter), the pH reached 5.75 for the control (W) sample and 5.63 for the Z treatment, with no significant difference (*p* > 0.05) between the two treatments. After day 4, the pH increased in the W and Z treatments due to the production of alkaline nitrogenous compounds formed through the decomposition of proteins. At the same time, due to the growth of LAB, which produce lactic acid, the pH is expected to decrease. In reality, the recorded pH value is the net result of the above two opposing phenomena. On day 10 of storage, the respective pH values were 5.58 for the C treatment and 5.64 for the CZ treatment, with no significant differences (*p* > 0.05) between the two. A significant difference (*p* < 0.05) was recorded between treatments W and Z and treatments C and CZ. The reduction in pH between days 2 and 8 is most probably due to the initial acidification caused by the addition of the chitosan/acetic acid solution. The present pH values are in agreement with those for beef meat reported by Castrica et al. [6], Chounou et al. [14], Hoa et al. [15], and Jiao et al. [32].

Color is the primary sensory parameter that the consumer evaluates when purchasing raw meat, indicating the degree of product freshness. The values of the color parameters L*, a* and b* are given in Table 1a–c. In addition to above individual color parameters, the total color difference (ΔE) was calculated using the following formula [33]:

ΔE = (ΔL*2 + Δa*2 + Δb*2)

A first observation to be made is that the initial L*, a*, and b* values are typical for good-quality and well-preserved raw beef meat 2 to 3 days post slaughter [34]. L* values (Table 1a) for the W and Z treatments showed a reducing pattern after day 2 of storage without significant differences (*p* > 0.05). On the other hand, the corresponding values for treatments C and CZ remained constant until day 4 and subsequently increased (*p* < 0.05) until the end of storage, also without significant differences (*p* > 0.05) between the two. According to Lawrie [35], low pH values, such as those of chitosan, on the surface of meat show a higher reflectance and thus higher L* values than those of untreated raw meat. a* values (Table 1b), related to the degree of oxymyoglobin oxidation to metmyoglobin, showed a decreasing pattern for all treatments with storage time. This decreasing trend, however, was steeper in the case of the W and Z treatments compared to that of the C and CZ counterparts. The above results support the hypothesis that chitosan preserves the red color of meat by acting as a chelating agent which binds Fe^2+^ ions, resulting in the inhibition of the oxidation of Fe^2+^ to Fe ^3+^ [34]. The differences between treatments W and Z were insignificant (*p* > 0.05) until day 6 of storage. The same holds for treatments C and CZ, in which statistically significant differences (*p* < 0.05) were noted between the two only toward the end of storage (days 8 and 10). The changes in the color parameter b* shown in Table 1c indicate a reduction in the degree of yellow for all treatments during storage. Yet, this reduction is steeper in treatments W and Z compared to that for treatments C and CZ. The degree of yellowness has been shown to be related to the phenomenon of oxidation [36]. As chitosan exhibits antioxidant as well as antimicrobial activity, it is evident that the chitosan dip maintains the color parameter b* values, as it did with the color parameter a* values.

With regard to the effect of the absorbent pad, the present results on color are in general agreement with those of Castrica et al. [6] and Fernandez et al. [25]. With regard to the effect of chitosan, the present results are in general agreement with those of Chounou et al. [14], Karakosta et al. [8], and Hoa et al. [15,19].

**Figure 3:**
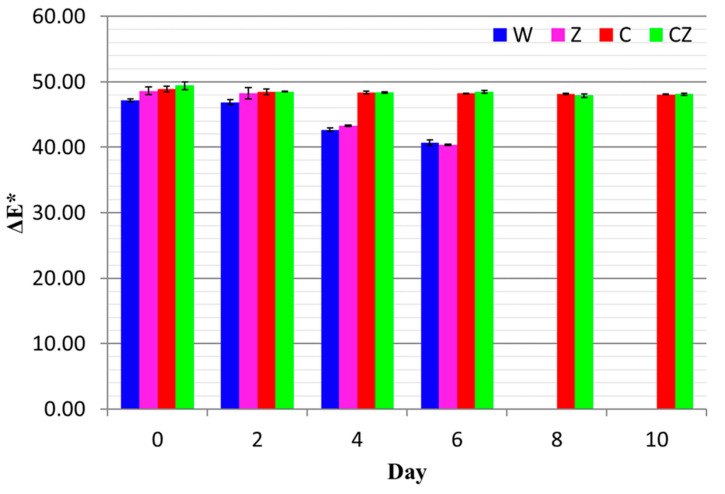
ΔE values of beef treatments as a function of storage time. W—control; Z—AgIon^®^ pad; C—chitosan; CZ—chitosan + AgIon^®^ pad.

Figure 3 shows the ΔE values for all treatments as a function of storage time.

For treatments W and Z, the ΔE values decreased from ca. 48 to 40.5 on day 6 of storage (*p* < 0.05), while the ΔE values for treatments C and CZ decreased from ca. 49 to 48 (*p* > 0.05) on day 10 of storage, indicating the protective effect of chitosan on meat color. According to Mokrzycki et al. [37], changes in the visual color of samples can only be clearly identified by an observer if ΔE* > 3.5. Such a total color difference was noted only in the W and Z treatments.

**Figure 4:**
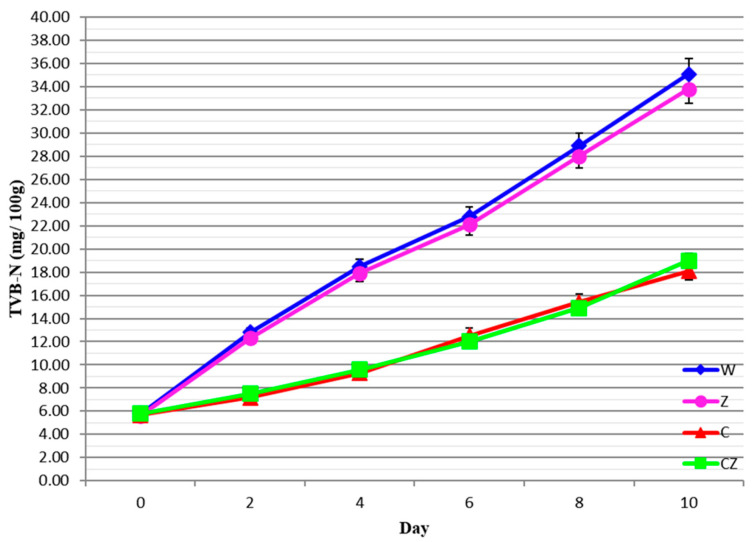
TVBN values of beef treatments as a function of storage time. W—control; Z—AgIon^®^ pad; C—chitosan; CZ—chitosan + AgIon^®^ pad.

TVB-N is often used as a biochemical marker of meat spoilage because of its positive association with both microbiological growth and proteolytic enzyme activity responsible for meat spoilage [38]. TVB-N includes ammonia, dimethyl amine, trimethylamine and other amino acid decarboxylation products. High TVB-N values are indicative of spoiled meat. The following guidelines have been proposed for fresh meat: TVB-N < 15 mg/100 g (National Food Safety Standard) [39], TVB-N < 20 mg/100 g (Korean Ministry of Agriculture and Forestry) [40], or TVB-N < 40.3 mg/100 g (Stephan et al.) [41]. It is important to note, however, that there is no direct comparison between TVB-N data and other measures of beef freshness [42]. TVB-N results for all treatments are shown in Figure 4.

The initial value for TVB-N was ca. 5.8 mg/100 g, increasing for all treatments with storage time. For treatments W and Z, TVB-N reached 18.5 and 17.9 mg/100 g on day 4 of storage, with no significant differences (*p* > 0.05) between the two treatments. On day 10 of storage, TVB-N recorded high values of 35.1 and 33.8 mg/100 g corresponding to unacceptable products. The respective TVB-N values for treatments C and CZ were 18.1 and 19.0 mg/100 g on day 10 of storage, with no significant differences (*p* > 0.05) between the two. Based on the proposed limit for TVB-N of 15–20 mg/100 g, it is suggested that acceptable quality of beef meat was maintained until day 4 for treatments W and Z and 10+ days for treatments C and CZ.

The present results for TVB-N are in general agreement with Castrica et al. [6], Cheng et al. [16], Hoa et al. [15], Duran and Kahve [2], Zou et al. [17], and Frank et al. [43] considering the differences in treatments applied to beef meat and the different temperatures used during meat storage.

### 3.3. Sensory Evaluation

**Figure 5:**
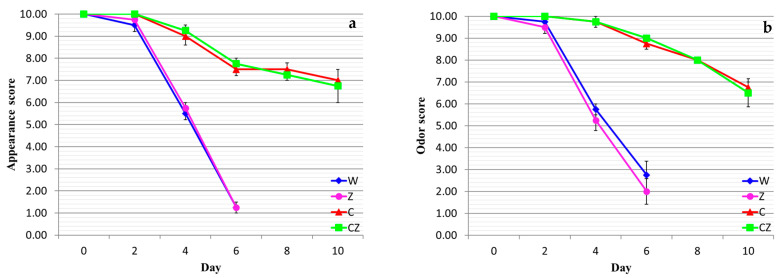
Sensory evaluation of raw beef treatments as a function of storage time: (**a**) appearance, (**b**) odor. W—control; Z—AgIon^®^ pad; C—chitosan; CZ—chitosan + AgIon^®^ pad.

The results of the sensory evaluation of raw meat are shown in Figure 5.

**Figure 6:**
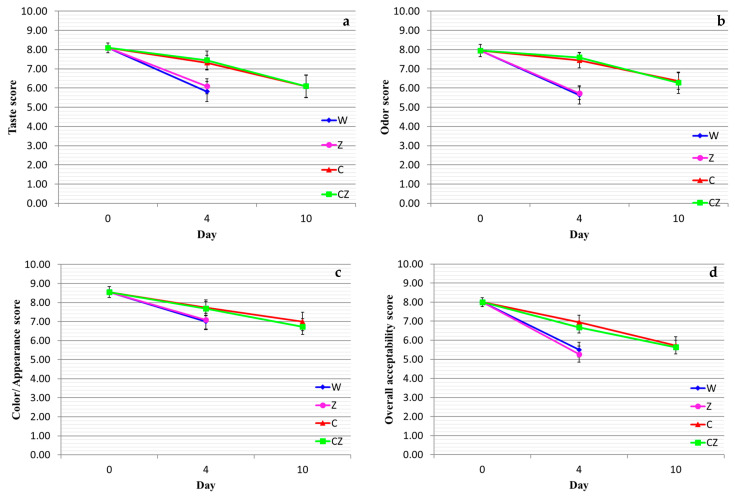
Sensory evaluation of cooked beef treatments as a function of storage time: (**a**) taste, (**b**) odor, (**c**) color/appearance, (**d**) overall acceptability. W—control; Z—AgIon^®^ pad; C—chitosan; CZ—chitosan + AgIon^®^ pad.

With regard to both appearance and odor, treatments W and Z displayed lower acceptability on day 4 of storage (i.e., day 7 after slaughter), while treatments C and CZ scored above 6 even after 10 days of storage. At a given sampling day, the differences between treatments W and Z and between treatments C and CZ were insignificant (*p* > 0.05), while the differences between the former set of values and the latter were statistically significant (*p* < 0.05). Both appearance and odor attributes were equally sensitive with regard to the degree of product acceptability. It is clear that the chitosan dip inhibited the growth of spoilage microorganisms, substantially increasing the shelf life of the raw products. The explanation for the negligible effect of the antimicrobial pad is the same as that already previously given. The results of the sensory evaluation of cooked meat are shown in Figure 6.

With regard to taste of the cooked meat (Figure 6a), treatments W and Z received a score of ca. 5.5–6.0 on day 4 of storage (i.e., day 7 after slaughter), while treatments C and CZ received a score of 6.0 on day 10 of storage. With regard to odor (Figure 6b), treatments W and Z received a score of ca. 5.0–5.5 on day 4 of storage (i.e., day 7 after slaughter), while treatments C and CZ received a score of 6.0–6.5 on day 10 of storage. With regard to color/appearance (Figure 6c), treatments W and Z received a score of ca. 7 on day 4 of storage (i.e., day 7 after slaughter), while treatments C and CZ received a score of 6.7–7.0 on day 10 of storage. For all three sensory attributes (color, taste and odor), there were no significant differences (*p* > 0.05) between treatments W and Z and between treatments C and CZ, while differences between the former set of values and the latter were statistically significant (*p* < 0.05). Of the three sensory attributes, taste and odor proved equally sensitive sensory properties with regard to the degree of product acceptability. Finally, according to the overall acceptability results (Figure 6d) treatments W and Z received an overall score of 5+ on day 4 of storage (i.e., day 7 after slaughter), while treatments C and CZ received an overall score of 5.6–5.8 on day 10 of storage.

It should be noted that the sensory evaluation results were in excellent agreement with those of the microbiological analysis (TVC) and physico-chemical analysis (TVB-N). The sensory evaluation results of the present study are in general agreement with those of Cheng et al. [16] for fresh beef cuts and Karakosta et al. [8] for fresh buffalo meat, considering the differences in treatments applied to meat and the different temperatures used during meat storage.

## 4. Conclusions

The present work has shown that based on sensory, microbiological, and physico-chemical data, a shelf life of 4 + 3 days (including time between slaughter and the beginning of shelf-life experiment) was achieved for treatments W and Z. The respective shelf life of treatments C and CZ was at least 10 + 3 days. This study also confirmed the preliminary finding of other studies that indicated that the Ag^+^-substituted zeolite does not exert a significant preservation effect when in direct contact with beef meat and that the combined preservative effect of treatments C and CZ was solely attributed to the chitosan coating.

## Figures and Tables

**Table 1:** Color parameters L*, a*, and b* of raw beef during refrigerated storage at 5 °C as a function of storage time.

Treatment		Day				
		0	2	4	6	8
(**a**)						
L*	W	41.12 ± 0.33	41.27 ± 0.41	39.42 ± 0.28	36.69 ± 0.26	-
		a A	a A	a B	a C	
	Z	41.21 ± 0.55	41.63 ± 0.59	40.05 ± 0.55	35.58 ± 0.21	-
		a A	a AB	a AB	a C	
	C	42.88 ± 0.53	43.53 ± 0.74	43.78 ± 0.457	44.15 ± 0.30	44.74 ± 0.14
		a A	b AB	b AB	b ABC	a BC
	CZ	42.19 ± 0.77	42.45 ± 0.12	43.55 ± 0.60	44.08 ± 0.51	43.81 ± 0.40
		a A	b AB	b ABC	b BC	a AB
(**b**)						
a*	W	20.84 ± 0.26	19.82 ± 0.06	13.86 ± 0.23	16.93 ± 0.57	-
		a A	ab A	a B	a C	
	Z	23.12 ± 0.14	21.90 ± 0.61	13.64 ± 1.47	18.23 ± 0.13	-
		a A	a A	a C	a Δ	
	C	20.95 ± 1.93	19.03 ± 0.96	18.09 ± 0.60	16.88 ± 0.51	14.85 ± 0.23
		a A	b AB	ab AB	a AB	a ABC
	CZ	23.20 ± 0.25	21.09 ± 0.09	18.66 ± 1.64	17.65 ± 0.74	16.74 ± 0.33
		a A	ab AB	b ABC	a C	b CΔ
(**c**)						
b*	W	10.00 ± 0.22	10.13 ± 0.15	8.57 ± 0.16	4.65 ± 0.22	-
		a A	ab A	a B	a C	
	Z	11.29 ± 0.19	10.81 ± 0.23	9.13 ± 0.16	5.59 ± 0.38	-
		b A	a A	a B	b C	
	C	10.37 ± 0.41	9.41 ± 0.53	9.66 ± 0.11	9.60 ± 0.17	9.55 ± 0.29
		ab A	b A	b A	c A	a A
	CZ	11.01 ± 0.21	10.25 ± 0.06	9.71 ± 0.05	9.51 ± 0.12	9.67 ± 0.11
		ab A	ab B	b B	c B	a B

Each value represents the mean value as the analysis was run in triplicate for each replicate (*n* = 2 × 3 = 6). The same lowercase indicator (a, b, c) suggests a statistically (*p* > 0.05) insignificant difference for the values along the same column, whereas the same uppercase indicator (A, B, C) suggests a statistically (*p* > 0.05) insignificant difference for the values along the same row.

## Data Availability

The original contributions presented in the study are included in the article. Further inquiries can be directed to the corresponding author.
